# Editorial: Nitrogen metabolism in crops and model plant species

**DOI:** 10.3389/fpls.2024.1502273

**Published:** 2024-10-08

**Authors:** Enrique Ostria-Gallardo, Luisa Bascuñán-Godoy, Néstor Fernández Del-Saz

**Affiliations:** ^1^ Laboratorio de Fisiología Vegetal, Departamento de Botánica, Universidad de Concepción, Concepción, Chile; ^2^ Department of Plant Physiology, University of Granada, Granada, Spain

**Keywords:** assimilation, transport, development, nutrition, nitrogen use efficiency, omics, biostimulants, stable isotopes

## Introduction

Nitrogen (N) is a critical macronutrient that influences various physiological, molecular, and metabolic processes in plants, playing a critical role in determining plant growth, crop yield, and environmental sustainability (O’Brien et al., 2016; The et al., 2021). However, excessive or inefficient use of nitrogen fertilizers has raised concerns regarding environmental degradation, including soil acidification and nitrogen leaching (Qasim et al., 2020; Dimkpa et al., 2020). This is especially pertinent as global demand for crop productivity continues to rise in conjunction with increased environmental stressors, both biotic and abiotic, driven by climate change (Coskun et al., 2017). This Research Topic brings together a collection of studies from molecular and physiological responses of either crops or model plants to large-scale agroecological solutions, reflecting the multidimensional efforts to optimize nitrogen use for both crop productivity and environmental sustainability.

## Molecular and genetic regulation of nitrogen uptake and signaling

A comprehensive review by Xu et al. examines the NRT2 family of nitrate transporters in *Arabidopsis*, detailing the roles of specific family members, such as NRT2.1, NRT2.4, and NRT2.5, in nitrate uptake under varying nitrogen conditions. By exploring how these transporters function within broader biological processes—including nitrogen remobilization and plant-microbe interactions—the authors provide a roadmap for future research aimed at enhancing NUE through genetic modification and selective breeding.

Similarly, Svietlova et al. investigate how glutamine, a key nitrogen compound, regulates nitrate transporter genes in *Arabidopsis thaliana*, particularly focusing on NRT2.4. Their research reveals that glutamine serves as both a nitrogen source and a signaling molecule, modulating the expression of nitrate transporters under nitrogen starvation conditions. This study underscores the importance of glutamine in nitrogen signaling and highlights potential pathways for optimizing nitrogen uptake at the molecular level.

Pélissier et al. further expand the discussion of nitrogen uptake by analyzing gene regulatory networks in rice, a staple crop. Their research identifies key transcription factors that govern nitrate and ammonium uptake, providing insights into the genetic control of nitrogen metabolism. This work not only deepens our understanding of nitrogen signaling but also highlights potential targets for improving nitrogen efficiency in crop production.

## Metabolic pathways and nitrogen assimilation

The metabolic regulation of nitrogen in plants is another focus of this Research Topic. Hu et al. explore the physiological mechanisms that enhance ammonium tolerance in wheat. Their findings demonstrate that glucose metabolism plays a critical role in supporting ammonium assimilation, ultimately reducing ammonium toxicity and promoting nitrogen uptake. This work offers potential strategies for developing wheat varieties that are more efficient in nitrogen assimilation, particularly under conditions of nitrogen stress.

In maize, Feng et al. investigate the interactions between nitrogen application and carbohydrate metabolism during grain filling. Their study demonstrates that an optimal nitrogen rate significantly enhances enzymatic activity related to both nitrogen and carbohydrate metabolism, improving grain quality and yield. These findings offer practical insights into nitrogen management strategies aimed at optimizing both metabolic efficiency and crop productivity.

The role of symbiotic relationships in nitrogen management is critical to reducing reliance on chemical fertilizers and improving agricultural sustainability. Alquichire-Rojas et al. introduce an exciting avenue of nitrogen management in *Chenopodium quinoa* through plant symbiosis with endophytic insect pathogenic fungi. These fungi promote nitrogen transfer from the soil to the plant, improving carbon storage, photosynthesis, and overall plant growth. Such symbiotic interactions represent promising tools for increasing nitrogen efficiency in agricultural systems, providing a more sustainable alternative to conventional fertilizers.

## Nitrogen management in horticultural and field systems

Zhang et al. focus on the role of nitrogen in metabolic shifts within pepper fruits, particularly how nitrogen influences carotenoid biosynthesis and fruit quality. Their research demonstrates that moderate nitrogen application optimizes pigment production, yield, and fruit quality, offering valuable guidance for nitrogen management in horticultural systems where quality is paramount.

The tight relationship between nitrogen and carbon is addressed in Feng et al. who explored the effects of nitrogen on carbohydrate metabolism in waxy maize, emphasizing the importance of nitrogen management during grain filling. Their findings show that appropriate nitrogen application enhances both starch and protein accumulation in grains, improving crop quality and performance. This research emphasizes the critical role of nitrogen management strategies in field crops.

## Water management and agroecosystem approaches

Chen et al. present a study on the impact of shallow wet irrigation (SWI) in paddy fields, addressing the persistent issue of nitrogen leaching in rice cultivation. Their work shows that SWI reduces nitrogen leaching by fostering beneficial microbial communities that enhance nitrogen fixation and rice yield. The integration of water management practices with nitrogen use efficiency highlights the potential for holistic agroecological approaches to improve both productivity and sustainability in cropping systems.

Tang et al. take a technological approach to nitrogen management, developing a precision fertilization model for durian cultivation using the IM-RBNNA algorithm. This data-driven tool provides accurate predictions of soil nutrient levels, optimizing nitrogen application and reducing fertilizer waste. The integration of advanced technology in fertilization strategies underscores the importance of precision agriculture in achieving higher yields while minimizing environmental impact.

## Perspective: toward an integrated approach for nitrogen use efficiency

The papers in this Research Topic illustrate the complexity of nitrogen metabolism and management, from molecular signaling mechanisms to agroecosystem applications. As global agricultural systems face increasing pressure to produce more food with fewer resources, the insights from these studies will play a pivotal role in guiding the next generation of sustainable nitrogen management practices.

**Figure 1:**
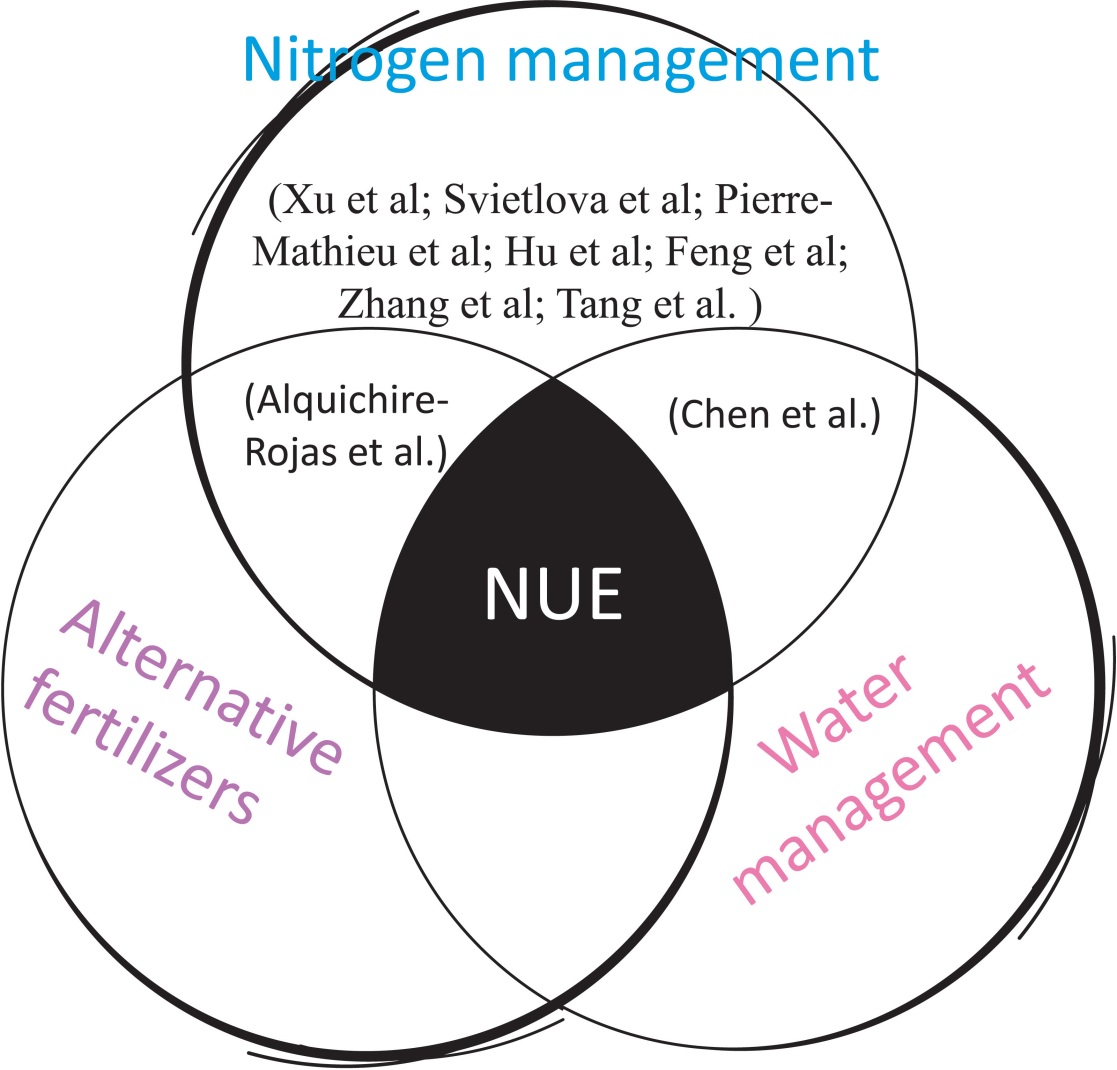
A Venn diagram showing the different studies belonging to this Research Topic, and three strategies (nitrogen assimilation, water management and alternative fertilizers), proposed by their authors, to improve NUE. These strategies require the integration of molecular, metabolic, and genetic approaches, as well as the use of soil microorganisms, and precision agriculture techniques.

By integrating molecular biology, metabolic regulation, microbial symbiosis, precision agriculture, and innovative water management (**Figure 1**), these contributions pave the way for more efficient and sustainable agricultural systems for a world experiencing an accelerated climate change.
